# Quorum-quenching for bacterial pathogen control and health management in aquaculture: mechanisms, applications, current status and future prospects

**DOI:** 10.3389/fsysb.2026.1861713

**Published:** 2026-06-04

**Authors:** Prapti Sudan, Anand Kumar, Neelesh Kumar, Satya Narayan Parida, Ajaya Kumar Rout, Prabjeet Singh, Partha Sarathi Tripathy, Bijay Kumar Behera, Pramod Kumar Pandey, Anuj Tyagi

**Affiliations:** 1 College of Fisheries, Guru Angad Dev Veterinary and Animal Sciences University, Ludhiana, Punjab, India; 2 College of Fisheries (Datia), Rani Lakshmi Bai Central Agricultural University, Jhansi, Uttar Pradesh, India; 3 Farm Science Centre (Tarn Taran), Guru Angad Dev Veterinary and Animal Sciences University, Ludhiana, Punjab, India; 4 National Fisheries Development Board, Department of Fisheries, Government of India, Hyderabad, Telangana, India

**Keywords:** antimicrobial resistance, aquaculture, bacterial pathogens, infectious diseases, quorum-quenching, quorum-sensing

## Abstract

The aquaculture sector has grown rapidly in recent years, and it now has a significant impact on global food and nutritional security. Infectious disease outbreaks in aquaculture cause significant losses and also threaten the sustainability. Despite the antimicrobial resistance (AMR), environmental contamination and consumer safety concerns, conventional disease control strategies still rely heavily on chemical antimicrobials. Thus, alternative, eco-friendly disease management approaches are urgently needed. This review describes quorum-quenching (QQ) as an innovative health management approach in aquaculture. Quorum-sensing (QS) is a communication system used by bacteria that depends on cell density. QS regulates gene expression, virulence factor production, and biofilm formation in key aquaculture pathogens, including *Vibrio*, *Aeromonas*, *Pseudomonas*, *Edwardsiella*, and *Flavobacterium* species. QQ disrupts QS signal networks through enzymatic degradation, competitive receptor inhibition, or suppression. In addition to discussing QS signal molecules and regulatory pathways, this review summarizes and critically evaluates enzymatic and non-enzymatic QQ mechanisms, including lactonases, acylases, oxidoreductases, and small-molecule inhibitors. Practical applications of QQ in aquaculture, including QQ probiotics (QQPs), functional feeds, water treatment systems, biofilm control and host immune modulation, have also been highlighted. While QQ offers a promising alternative to traditional antimicrobials. Translating this technology to field scale applications presents critical challenges. Specifically, the broad-spectrum nature of some QQ agents raises concerns regarding target specificity and the unintended disruption of beneficial native microbiomes. Furthermore, the long term environmental ecological impacts of introducing foreign QQ enzymes or organisms into complex aquatic ecosystems requires rigorous evaluation. Crucially, QQ exerts less selective pressure than bactericidal agents, the potential for pathogens to develop resistance mechanisms such as receptor modification or signal bypassing must be carefully monitored. This study focuses on research pertinent to quorum-quenching in aquaculture systems and is based on a narrative synthesis of peer-reviewed literature that was obtained from major scientific databases. Mechanistic findings, aquaculture application, and translational possibilities in field settings are highlighted.

## Introduction

The rapidly expanding aquaculture sector has been playing an important role in food and nutritional security for a growing global population. Currently, capture fisheries production has peaked, and more than half of global fish production comes from aquaculture. In the year 2022, global aquaculture production was 94.4 million tonnes (FAO, 2024). In addition to meeting food and nutritional needs, the aquaculture sector also provides employment for millions of people in developing and developed nations. The remarkable growth of the aquaculture sector is frequently hindered by infectious disease outbreaks, which remain the primary constraint to sustainable production (Stentiford et al., 2012; Thakur et al., 2023). The aquaculture industry has experienced an estimated 21% production loss, resulting in annual losses of US$ 6 billion. The shrimp industry itself has incurred losses of approximately US$10 billion since 1990 (WorldBank, 2014). Major bacterial pathogens in aquaculture are *Vibrio* spp. (*Vibrio harveyi, Vibrio parahaemolyticus, V. vulnificus* and *V. anguillarum*), *Aeromonas* spp. (*Aeromonas hydrophila, Aeromonas salmonicida, Aeromonas veronii* and *A. dhakesnis*) *Pseudomonas* spp. (*Pseudomonas aeruginosa* and *Pseudomonas fluorescens*) and *Edwardsiella tarda.* These pathogens cause more than 60% of all bacterial disease outbreaks in aquaculture. Several interconnected factors, like very high stocking density, poor water quality, stress, and environmental degradation, create ideal conditions for pathogen growth and transmission in aquaculture (Hoseinifar et al., 2018; Toranzo et al., 2005).

The heavy reliance on chemical antimicrobials for controlling bacterial diseases has led to the development and spread of antimicrobial resistance (AMR) (Milijasevic et al., 2024; Sudan et al., 2023). Bacteria found in aquatic environments can carry antimicrobial resistance (AMR) genes, which may be transferred to other bacterial pathogens (Tyagi et al., 2019). Moreover, antimicrobial residues in fish products can harm human health, and their maximum residual limits are strictly regulated in major import markets. These AMR and consumer safety concerns have necessitated alternative approaches to disease management (Bacanlı, 2024). In this context, targeting the bacterial communication system Quorum-sensing (QS) has become a promising strategy. Conventional antimicrobials either kill bacteria or inhibit their growth, creating significant selective pressure on bacterial populations. Quorum-quenching (QQ) interrupts QS pathways, attenuating bacterial pathogenicity without directly killing the bacteria. In this approach, pathogen virulence is mitigated, and compared to bactericidal drugs, QQ may impose less selective pressure since it affects bacterial communication rather than survival. However, it is impossible to completely rule out the potential of adaptive resistance, signal bypass mechanisms, or compensation pathways, and long-term uses need to take these hazards into account (Chu et al., 2014; Vadassery and Pillai, 2020).

This review summarizes and evaluates QQ as an innovative health management strategy. In addition to examining the QS mechanism in major aquaculture pathogens, we have explored both enzymatic and non-enzymatic QQ strategies to disrupt QS communication systems. The current knowledge of natural and synthetic QQ compounds, their sources, and mechanisms of action has also been covered in detail. Practical applications of the QQ concept in aquaculture, including quorum-quenching probiotics (QQPs), feed additives, and water treatment approaches, have also been comprehensively covered. The review also covers molecular and physiological effects of QQ, implementation challenges, and economic considerations. We have also identified knowledge gaps and future research directions to promote QQ as an eco-friendly health management strategy for sustainable aquaculture.

While numerous present reviews have far mapped the biochemical and molecular pathways of QS and QQ, a significant gap remains in translating this fundamental microbiological theory into scalable, practical interventions for aquaculture farms. Therefore, the exclusive contribution of this review lies in critically bridging the theoretical mechanisms of QQ with its real-world applications in aquaculture. We expansively examine how the targeted molecular disruption of QS communication *via* enzymatic and non-enzymatic approaches is practically engineered into field-ready tools. Specifically, we investigate the translation of these biological concepts into concrete health management strategies, including quorum-quenching probiotics, functional feed additives, and integrated water-treatment biofilters. By evaluating the molecular physiological effects of QQ together with farm-level implementation challenges and economic feasibility (Bacanlı, 2024), this review follows a narrative review approach to synthesise current knowledge on QQ in aquaculture systems. The majority of the studies considered were published between 2000 and 2025. Peer-reviewed original research publications were prioritized, especially those that addressed aquatic systems and aquaculture diseases. For conceptual comprehension, other review papers were examined. The selection of studies was focused on their practical usefulness in aquaculture settings, pathogen management, and relevance to QQ mechanisms. No formal systematic screening or meta-analysis was carried out.

## Concept of quorum-sensing

Through the QS communication system, bacterial populations coordinate their collective behaviors according to cell density (Deo et al., 2025; Zulfiqar and Akan, 2025). It operates by synthesizing, releasing, and detecting small signaling molecules known as autoinducers. At the threshold level, these signaling molecules bind to receptors and modulate regulatory pathways across the microbial community. Although first identified in the bioluminescent marine bacterium *Vibrio fischeri*, QS has now been recognized in a variety of bacterial taxa, including significant aquaculture pathogens (Bhomkar and Naik, 2025). The core components of QS systems are: (i) signal synthases that produce autoinducers, (ii) receptors and (iii) regulatory networks that control target genes (Kachhadiya and Georrge, 2025). This density-dependent regulation allows bacteria to synchronize biofilm formation, virulence factor secretion, motility, and bioluminescence behaviours. QS contributes to pathogen survival and disease development by regulating virulence, host colonization, and immune response. It also influences nutrient cycling and microbial interactions, which are critical for ecosystem health (Bhomkar and Naik, 2025).

## Quorum-sensing signal molecules

### N-acyl homoserine lactones (AHLs)

AHLs are extensively studied QS signal molecules. In Gram-negative bacteria, AHLs regulate population-wide behaviours in the environment (van Aalst et al., 2025). AHL structure usually consists of a homoserine lactone ring, which is connected to an acyl chain (C4–C18) through an amide bond. Sometimes, signal specificity is also enhanced by hydroxy, oxo, or unsaturated substitutions at the C3 position (Poonguzhali et al., 2025). AHLs are produced by LuxI-type enzymes and are recognized by LuxR-type transcriptional regulators. Once AHLs reach their threshold levels, they bind to LuxR homologs and activate QS-regulated gene expression. This allows the bacterial populations to synchronize complex behaviors, including biofilm formation, motility, virulence factor production, and antibiotic resistance (Kumar et al., 2022). In pathogenic *A*. *hydrophila* and *Vibrio* species, specific AHLs, such as C4-HSL and C6-HSL, are strongly associated with biofilm formation and virulence (Nagar et al., 2015). Moreover, AHL-mediated signaling regulates the secretion of proteases, hemolysins, and siderophores, which enhance pathogenicity (Table 1). Recent evidence indicates that disruption of AHL-based signaling can weaken bacterial virulence and reduce disease severity. Consequently, AHL signaling disruption strategies such as QQ enzymes and probiotic-mediated interference are being actively explored to mitigate bacterial pathogenicity in aquaculture (Langlois et al., 2021).

**TABLE 1:** Major AHL-mediated QS signals and their functions in important aquaculture pathogens.

QS signal gene	Pathogen	Regulated functions	References
*ahyI*, *ahyR* (AHL-based QS)	*A. hydrophila*	Turmeric oil (sub-MIC) inhibited QS-regulated virulence phenotypes, downregulated *aerA*, *ahyI*, and *ahyR* transcription; reduced fish mortality; alleviated renal injury	Dong et al. (2025)
*ahyI*, *ahyR*	*A. hydrophila*	Hemolysin, lipase, protease production; swarming motility; biofilm formation	Payam et al. (2025)
AHLs (degraded by isolates)	*P. aeruginosa*	EPS secretion and biofilm formation inhibited by AHL-degrading bacteria (*Bacillus cereus*, *Paenibacillus polymyxa*, *Mesorhizobium thiogangeticum, Microbacterium paraoxydans*)	Tomy and Yasarla (2025)
*ahyI* (AHL-based QS)	*A. veronii* Z12	Regulated intestinal colonization *via* chemotaxis genes; deletion of *ahyI* or *cheA* reduced chemotaxis, colonization, and niche competition	Li et al. (2024)
AHLs, AI-2	*A. salmonicida* GMT3	Spoilage and virulence: protease/lipase, motility, biofilm, hemolysin, T2SS/T6SS, antibiotic resistance	Liu et al. (2024)
C4-HSL, C6-HSL	*Ochrobactrum* sp. LC-1	Biomass increase, EPS and protein secretion, enhanced adhesion; synergistic biofilm promotion in quinoline degradation	Gao et al. (2023)
Diverse AHLs (LuxI/LuxR systems)	*P*. *aeruginosa, Chromobacterium violaceum, Agrobacterium tumefaciens, E*. *coli*	Molecular diversity of AHL synthases and receptors; regulation of QS-mediated gene expression	Kumar et al. (2022)
C4-HSL, C6-HSL	*Aeromonas* sp. A-L2	Induced swarming; C6-HSL stimulated EPS secretion; C4-HSL/C6-HSL enhanced biofilm formation	Gao et al. (2020)
AHLs; QS inhibitors (HepS-AHL)	*A. salmonicida*	Protease, lipase, pigment, A-layer production; QSI (HepS-AHL) reduced protease production up to 10-fold	Rasch et al. (2007)

### Autoinducer-2 (AI-2)

AI-2 is a highly conserved QS signal molecule. Since AI-2 is produced by both Gram-negative and Gram-positive bacteria, it functions as a “universal language” for communication between different species. AI-2 QS proteins are distributed across 17 bacterial phyla (∼37% of genomes), with Gram-positive bacteria primarily producing AI-2 and Gram-negative bacteria responding to it. The AI-2 receptor CahR likely evolved from methyl-accepting chemotaxis proteins, linking QS to c-di-GMP signaling and bacterial adaptation (Liu et al., 2025). Unlike species-specific QS signals, AI-2 enables diverse microbial communities to coordinate behaviors such as biofilm formation, motility, virulence, and resource acquisition. It is enzymatically synthesized from 4,5-dihydroxy-2,3-pentanedione (DPD) by LuxS and exists in multiple interconvertible forms (Zhang et al., 2020). With the help LuxP and LsrB receptors bacteria detect AI-2 and trigger intracellular signaling cascades that regulate gene expression. AI-2 signaling influences microbial community dynamics through promotion of mixed-species biofilms, regulation of genetic competence, and facilitation of horizontal gene transfer. AI-2-mediated QS regulates pathogenic behaviors in *Vibrio* and *Aeromonas* species. AI-2-mediated signaling enables *Aeromonas veronii* to breach intestinal barriers, colonize gonads, induce tissue damage, and disrupt sex hormone balance in loaches (Niu et al., 2025).

### Other signaling molecules

Besides AHLs and AI-2, bacteria also produce several other QS signal molecules. Gram-positive bacteria primarily produce autoinducing peptides (AIPs). These short oligopeptides (5–17 amino acids) are often modified post-translationally to mediate cell-to-cell communication (Wadhwa and Kakkar, 2024). *Pseudomonas* quinolone signal (PQS) regulate virulence in *Pseudomonas aeruginosa* and related aquatic species (Long et al., 2025). Indole and its derivatives, derived from tryptophan metabolism, function as intra- and interspecies signals across diverse bacterial taxa. These molecules regulate biofilm formation, motility, and other virulence factors (Defoirdt, 2023). These diverse signaling molecules expand the repertoire of QS-mediated regulation in pathogenic bacteria.

## Quorum-sensing mechanisms in major aquaculture pathogens

### *Vibrio* species

**FIGURE 1:**
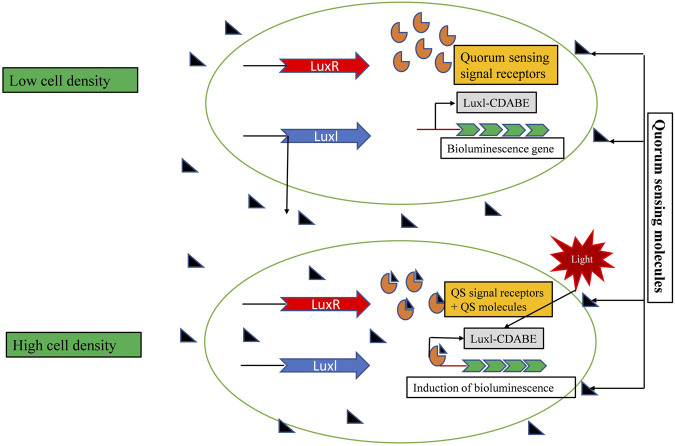
Cell-density–dependent induction of bioluminescence in *V. fischeri* (Prepared using Microsoft PowerPoint).

In *Vibrio* species, autoinducers (AIs) are key signaling molecules that regulate quorum-sensing, influencing vital functions like bioluminescence, virulence, motility, and biofilm formation (Girard, 2019). In *V*. *fischeri* QS revolves around three interlinked systems: the LuxI/LuxR, AinS/AinR, and LuxS/LuxP/Q pathways (Figure 1). The LuxI enzyme synthesizes 3-oxo-C_6_-HSL, which interacts with LuxR to activate the lux operon involved in bioluminescence. Meanwhile, AinS generates C_8_-HSL, which signals through AinR and also directly binds LuxR, though less effectively than 3-oxo-C_6_-HSL. A third signal, AI-2, generated by LuxS and detected *via* LuxP/Q, feeds into the same phosphorelay network, modulating LitR *via* LuxO and Qrr1 sRNA (LuxO phosphorylation at low density keeps LitR low; this lifts at higher density, activating LuxR and luminescence) (Verma and Miyashiro, 2013).

QS also regulates other functions in *V. fischeri*, including metabolic processes such as the acetate switch *via* AinS/LitR, motility (enhanced at low density through LuxO–Qrr1–LitR), and biofilm formation involving the SYP polysaccharide and cellulose. LitR for which AinS and LuxS are upstream regulators acts to inhibit cellulose transcription under specific conditions (Fung Brittany and Visick Karen, 2025). In *Vibrio cholerae*, the QS network responds to CAI-1 (*via* CqsA/CqsS) and AI-2 (*via* LuxS/LuxPQ). At low cell density, AphA is activated and HapR is repressed, promoting biofilm formation and virulence. With increasing AI levels, HapR is induced, leading to repression of biofilm formation and dispersal (Bridges and Bassler, 2019; Lin et al., 2024). A third QS pathway involves DPO and VqmA, which produces VqmR sRNA that post-transcriptionally represses AphA, further dampening virulence and biofilm genes (Herzog et al., 2019). Collectively, these *Vibrio* QS circuits integrate multiple AIs, phosphorelays, transcription factors, and sRNAs to finely regulate communal behaviour in response to cell density and environmental conditions.

### *Aeromonas* species

*Aeromonas* species deploy multiple QS mechanisms, most notably AI-1 (AHL-based) and AI-2 (LuxS-mediated), to regulate key behaviours such as biofilm formation, motility, virulence, and host colonisation.

### *AI-1 (AHL-dependent)* system

In *Aeromonas hydrophila* and *Aeromonas salmonicida*, QS relies on LuxI/LuxR homologs, designated AhyI/AhyR and AsaI/AsaR, respectively. These produces and detect N-(butanoyl)-L-homoserine lactone (C4-HSL) as the primary signal, with N-hexanoyl-HSL (C6-HSL) present as a minor autoinducer (Swift et al., 1997). During exponential growth, AhyI synthesizes C4-HSL, which binds AhyR to enhance ahyI transcription in an autoinduction loop, amplifying QS signalling. At the stationary phase, AhyR suppresses ahyI expression, shifting control to intercellular communication *via* accumulated AHLs (Talagrand-Reboul et al., 2017). AI-1 signalling in *Aeromonas* modulates biofilm maturation, extracellular protease production, and expression of Hcp and VgrG effectors of Type VI Secretion System (T6SS) (Khajanchi Bijay et al., 2010).

### *AI-2 (LuxS-mediated)* system

*Aeromonas* also encodes LuxS, producing the interspecies autoinducer AI-2. In *A. veronii*, LuxS mutation reduces adhesion to host tissues (e.g., erythrocytes, mucus) and lowers c-di-GMP levels. Complementation restores adhesion, suggesting that AI-2 positively regulates host colonization *via* modulation of c-di-GMP and QS regulators such as HapR and AphA (Li et al., 2023). Different strains can produce a range of AHLs. For instance, *A. veronii* strain LP-11 produces unusual molecules such as 3-o-OH-C8-HSL and C14-HSL, unlike the typical C4-/C6-HSLs found in other *Aeromonas.*

### *Pseudomonas* species

In fresh fish products, excessive loads of *Pseudomonas* can lead to their rapid spoilage. It is wise for Food Business Operators (FBOs) to consider its presence both in whole and prepared fish products. It is widely demonstrated that Gram-negative bacteria belonging to the Pseudomonadaceae family are one of the most active microbial populations in the production of proteolytic, lipolytic and saccharolytic enzymes (also extracellular ones). *P. aeruginosa* and *Pseudomonas fluorescens* use AHL-based QS circuits, primarily the LasI/LasR and RhlI/RhlR systems, to regulate genes involved in virulence, biofilm development, and antibiotic resistance. In aquaculture, their QS molecules can disrupt fish epithelial barriers and modulate immune responses, thereby increasing fish mortality. QS inhibition can curb biofilm formation, reduce pathogenicity, and facilitate the management of aquaculture diseases (Priya et al., 2023; Vanessa et al., 2024). The *P. aeruginosa* QS system consists of two complete circuits that involve acyl-homoserine lactone signals and a third system that uses quinolone signals. Together, these three QS circuits regulate the expression of hundreds of genes, many of which code for virulence factors. *P. aeruginosa* has become a model for studying the molecular biology of QS and the ecology and evolution of group behaviors in bacteria (Miranda et al., 2022).

### *Edwardsiella* species

*Edwardsiella ictaluri* and *Edwardsiella tarda* produce several AHLs and utilize the LuxS/AI-2 system for QS regulation. At high cell density or in stationary growth phases, QS pathways promote biofilm formation, virulence factors production, and metabolic adaptation. QS disruption through QQ bacteria slows disease progression and improves fish survival (Sun et al., 2020).

### *Flavobacterium* species

*Flavobacterium* is recognised as an important genus in the phylum Bacteriodetes and is considered a major mineralizer of organic matter. These bacteria have been found both free living and attached to organic aggregates, and can be associated with phytoplanktons. *It also* commonly associated with infections in stressed fish. The major concern regarding some members of this genus is their ability to cause disease across a variety of aquaculture settings. Fish infections caused by pathogenic *Flavobacterium* spp. are a major problem in the aquaculture industry worldwide, often resulting in significant economic losses. *Flavobacterium columnare*, the pathogen responsible for columnaris disease in freshwater fish, also utilizes quorum-sensing (QS) to manage biofilm development and virulence. However, QS systems of *Flavobacterium columnare* are less characterized than those of *Pseudomonas* or *Edwardsiella*. Recent studies have linked QS-mediated biofilm formation to phage resistance and disease severity (Kunttu Heidi et al., 2021).

## Quorum-quenching: concept, mechanisms and types

**FIGURE 2:**
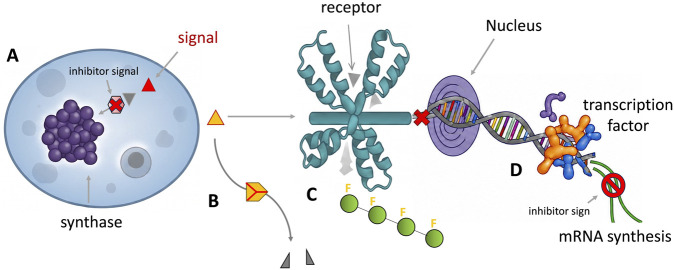
Quorum-quenching strategies **(A)** Signal biosynthesis inhibition by small molecules, **(B)** The breakdown of signaling molecules through enzymatic processes, **(C)** The suppression of signal reception by small molecules, and **(D)** Inhibition of signal transduction by small molecules (Prepared using Microsoft PowerPoint).

QQ refers to the process of interrupting QS. The concept of QQ involves biological, chemical, or physical interventions that interfere with QS. Reported mechanisms include enzymatic degradation of autoinducers by lactonases and acylases (Koch et al., 2014), signal mimicry through decoy molecules that competitively block receptors, and inhibition of autoinducer biosynthesis. Besides, chemical or physical factors can also destabilize the signals. In a broader sense, QQ strategies are grouped into two major approaches. In the first approach, the production, transport, or detection of QS signals is inhibited by small molecules. In the second approach, enzymes degrade signalling molecules and prevent microbial communication (Figure 2). By obstructing QS, QQ reduces bacteria’s ability to coordinate pathogenic or detrimental activities (Murugayah and Gerth, 2019).

### Molecular mechanisms and host-pathogen interactions during quorum-quenching

**FIGURE 3:**
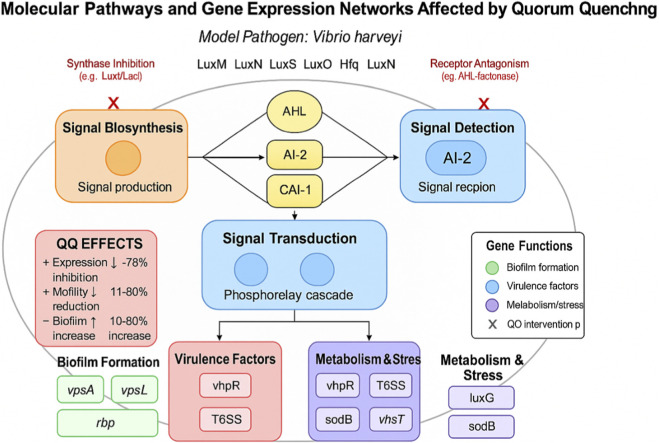
Molecular pathways and gene expression networks affected by QQ in *V. harveyi* (Prepared using Microsoft PowerPoint).

By targeting QS without directly inhibiting bacterial growth, QQ reduces selection pressure for antibiotic resistance. The molecular pathways and gene expression networks affected by QQ in *Vibrio harveyi* are illustrated in Figure 3. This figure highlights the multifaceted impact of QQ on bacterial virulence and host-pathogen interactions (Defoirdt et al., 2008; Shaheer et al., 2021).

AHL-lactonase treatment has been reported to result in up to 90% reduction in intestinal mucosa colonization by *V. anguillarum* under experimental conditions. During reduced colonization, bacteria were present as scattered individual cells rather than forming dense microcolonies. This effect was due to the downregulation of QS-regulated mucosal adhesins and mucin-binding proteins (Finlay and Falkow, 1997). Similarly, QQ treatment of *A*. *hydrophila* in Nile tilapia markedly decreased tissue damage and bacterial dissemination. In QQ treated fish, the pathogen caused minimal invasion and damage to the kidney, liver, and spleen (El-Bahar et al., 2019). Studies on *V*. *harveyi* demonstrated that AI-2-mediated QS was essential for its pathogenicity in *Artemia franciscana*. Disruption of AI-2 signaling suggests pathogenicity (Defoirdt et al., 2008). Moreover, QQ enhances host immune responses by reducing bacterial complement-resistance factors and increasing susceptibility to antimicrobial peptides.

### Enzymatic quorum-quenching

Enzymatic QQ utilizes specific enzymes to degrade or modify QS signal molecules (Rehman et al., 2022). These enzymes primarily target the predominant AHL QS signals in Gram-negative bacteria (Table 2). Three main classes of QQ enzymes have been characterized based on their mechanisms of action: AHL-lactonases, AHL-acylases, and AHL-oxidoreductases.

**TABLE 2:** Quorum-quenching enzymes with activity against major aquaculture pathogens.

Enzyme class	Source organism	Target pathogen(s)	Host species	Observed effects	References	Evidence category
AHL-acylases	*Ralstonia* spp.	Various Gram-negative bacteria	Aquatic species	Hydrolyzes long-chain AHLs, disrupting QS and associated virulence	Kalia et al. (2011)	Aquatic/Environmental relevance
	*Shewanella* sp.	*V. coralliilyticus*, *V. owensii*	Shrimp, bivalves	Decreased AHL levels, reduced virulence, diminished biofilm and protease activity, improved survival rates	Reina et al. (2021)	Direct aquaculture evidence
	*Stenotrophomonas maltophilia*	Opportunistic Gram-negative pathogens	Not specified	QQ *via* AHL degradation; potential to reduce virulence and antibiotic resistance	Bravo et al. (2025)	Clinical/Model organism evidence
AHL-lactonases	*Bacillus* sp. *B546*	*A. hydrophila*	Common carp, zebrafish	Reduced mortality; attenuated virulence; stable at moderate pH and temperature	Chen et al. (2010)	Direct aquaculture evidence
	*Bacillus* sp. *AI96*	*A. hydrophila*	Zebrafish	Decreased infection; stable at high temperature and alkaline pH; resistant to proteolysis	Cao et al. (2012)	Direct aquaculture evidence
	*B. licheniformis* DAHB1	*V. parahaemolyticus*	Indian white shrimp	Reduced colonization and infection; lowered mortality; broad AHL degradation	Vinoj et al. (2014)	Direct aquaculture evidence
	*Bacillus* sp. *QSI-1*	*A. hydrophila*	Goldfish (*Carassius auratus*)	Modified gut microbiota; reduced pathogen abundance	Zhou et al. (2016)	Direct aquaculture evidence
	*Kurthia huakuii*	*P. aeruginosa*	Not specified	Reduced biofilm formation and virulence factor production	Dong et al. (2018)	Model organism evidence
	*Psychrobacter* sp.	*S. marcescens*, *Vibrio* spp.	Fish pathogens	Effective degradation of short- and medium-chain AHLs	Packiavathy et al. (2021)	Aquatic/Environmental relevance
	*Bacillus* sp.	*A. hydrophila*	Fish (co-injection study)	Altered intestinal flora; >50% reduction in mortality	Peng et al. (2021)	Direct aquaculture evidence
AHL-oxidoreductases	*Rhodococcus erythropolis* W2	Various AHL-producing bacteria	Not specified	Modified AHL molecules *via* oxidation/reduction; disrupted QS signaling	Uroz et al. (2005)	Environmental relevance
	Metagenomic clones	*K. oxytoca*, *K. pneumoniae*	Not specified	Interfered with AHL and AI-2 signaling; reduced biofilm formation	Weiland-Bräuer et al. (2016)	Clinical/Model organism evidence

#### AHL-acylases

AHL-acylases hydrolyse the amide bond in AHLs and disrupt QS (Kusada et al., 2017). Most AHL-acylases are classified as part of the N-terminal nucleophile (Ntn) hydrolase family based on their structural characteristics. These enzymes typically form α/β heterodimeric folds with specialized substrate-binding pockets. For example, the hydrophobic pocket in PvdQ from *P*. *aeruginosa* enables recognition of long acyl chains, while overall structural diversity allows adaptation to different substrates and provides opportunities for protein engineering to enhance stability, activity, and specificity (Bokhove et al., 2010). These enzymes, also called amidases or amidohydrolases, cleave the lactone-acyl bond and act specifically on medium-to long-chain AHLs (C_10_–C_14_). The wide distribution of AHL-acylases in prokaryotes and eukaryotes suggests additional physiological functions beyond QS interference. Some AHL-acylases, like MacQ, also degrade β-lactam antibiotics, linking QQ with multidrug resistance mechanisms (Kusada et al., 2017).

#### AHL-lactonases

AHL-lactonases also degrade AHLs. Based on structural and catalytic features, AHL-lactonases are divided into four primary groups.

1. Metallo-β-lactamases (MBLs): MBLs have a conserved Zn^2+^-binding domain (HXHXDH). This domain is crucial for the catalytic activity of MBLs. Examples of MBLs are AiiA from *Bacillus* sp. 240B1 and AttM and AiiB from *Agrobacterium tumefaciens* C58 (Cai et al., 2018; Zhu et al., 2023).
2. Paraoxonases (PONs): PONs is found in vertebrates, particularly in mammals. PONs have lactonase activity, and these can hydrolyze a range of substrates, including AHLs. PON2 efficiently inactivates AHLs and exhibits aryl esterase activity. The expression of human PON1 in *Drosophila melanogaster* reduced the virulence of *P. aeruginosa* and modulated the gut microbiota (Draganov et al., 2005).
3. α/β hydrolase fold lactonases: These enzymes have a conserved catalytic triad consisting of a nucleophile, histidine, and an acidic amino acid. These enzymes demonstrate significant hydrolytic activity against different AHLs. AidH from *Ochrobactrum* sp. T63, AiiM from *Microbacterium testaceum* StLB037, and JydB from *Rhodococcus* sp. BH4 belong to this group (Mei et al., 2010).
4. Phosphotriesterase-like lactonases (PLLs): PLLs belong to the amidohydrolase family and can hydrolyze a variety of substrates, including AHLs. Examples are QsdA from *R. erythropolis*, GKL from *Geobacillus kaustophilus*, and GsP from *G. stearothermophilus.* These enzymes are known for their thermostability and broad substrate specificity (Zhu et al., 2023).

#### AHL-oxidoreductases

AHL-oxidoreductases oxidize or reduce AHLs, thereby inactivating them. These enzymes have been seldom reported and may target molecules beyond QS signals (Xu et al., 2024). BpiB09, an NADP-dependent reductase isolated from the soil metagenome, inactivated 3-oxo-C12-HSL by reducing its C3 carbonyl group. The expression of BpiB09 in *P*. *aeruginosa* downregulated QS-controlled virulence genes and alleviated *Caenorhabditis elegans* paralysis (Bijtenhoorn et al., 2011; Rodríguez-Urretavizcaya et al., 2024).

#### Non-enzymatic quorum-quenching

In non-enzymatic QQ, chemical compounds or biological molecules disrupt QS without enzymatic degradation of signal molecules (Hetta et al., 2024). These inhibitors interfere with bacterial communication by mimicking QS signals or binding QS receptors. Non-enzymatic QQ disarms pathogens without affecting essential bacterial processes, potentially exerting less selective pressure and delaying the development of resistance. Strategies include signal mimicry, in which compounds resembling natural QS signals competitively bind to receptors; receptor antagonism, in which small molecules or peptides block receptors without activating them; and signal sequestration, in which QQ agents bind QS signals to prevent receptor interaction. These compounds are often stable, easy to apply, and can act broadly across multiple QS systems, making them an alternative to conventional antibiotics (Alum et al., 2025).

#### Small molecule inhibitors

Small molecule inhibitors disrupt QS without degrading the signal molecules. These molecules either inhibit signal synthesis or compete with natural signals at receptor sites (Cimolato et al., 2025). Small molecule inhibitors of natural origin (flavonoids, ajoene, furanones, polyphenols) and synthetic origin (5-fluorouracil, azithromycin, aspirin, benzothiazoles) show QS inhibitory activity. In aquaculture, these inhibitors reduce virulence, biofilm formation, and motility in pathogens like *Vibrio* spp., *Aeromonas hydrophila*, and *Pseudomonas aeruginosa* (Gupta and Kumar, 2022; Srinivasan et al., 2025).

#### Natural quorum-sensing inhibitors

Natural quorum-sensing inhibitors (QSIs) are bioactive compounds derived from diverse species, particularly plants. The first natural product with QS inhibitory activity was brominated furanone produced by the marine alga *Delisea pulchra*. This compound structurally resembles AHLs and prevents their binding to LuxR-type receptors. It also inhibits the AI-2-mediated QS systems (Defoirdt et al., 2007). Coumarins also inhibit QS signaling pathways and decrease biofilm formation in clinically important pathogens. These findings highlight natural QSIs as viable candidates for sustainable antimicrobials (Reen et al., 2018). In zebrafish, citrus flavonoid hesperidin methylchalcone (HMC) inhibited *A. hydrophila* QS, resulting in reduced bacterial colonization and fish mortalities (Roshni et al., 2023).

Animal-derived QSIs have also been identified in diverse organisms and biological matrices. Mammalian paraoxonases (PONs), which play roles in ester and lactone detoxification, also hydrolyze AHLs. Sera from mice, goats, horses, and rabbits effectively inactivate the QS signal 3-oxo-C12-HSL (Dong and Zhang, 2005; Draganov et al., 2005). Additionally, dietary products like turkey patties, chicken breast, and cheese have been found to reduce AI-2 activity by 84%–99% (Lu et al., 2004).

#### Quorum-sensing inhibitors from marine organisms

**FIGURE 4:**
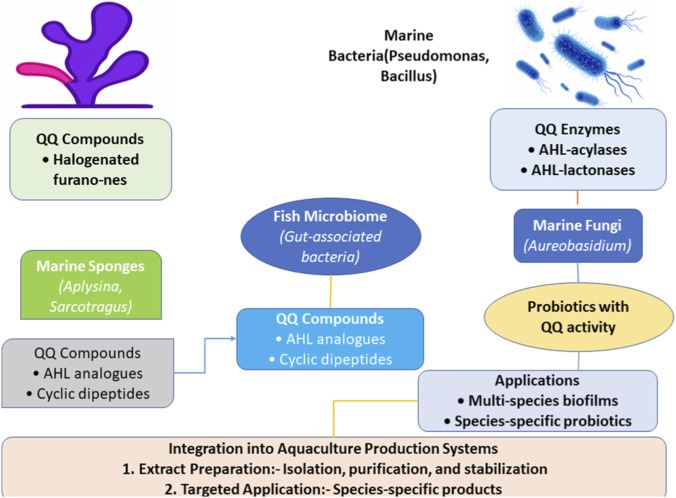
Marine-derived quorum quenching (QQ) compounds and their potential applications (Prepared using Microsoft PowerPoint).

Marine organisms are valuable sources of quorum-sensing inhibitors (QSIs) with diverse structural features and mechanisms of action (Figure 4). For instance, *Nocardiopsis dassonvillei* XG-8-1 produces novel α-pyrones (nocapyrones H, I, and M) that inhibit the expression of genes involved in QS in *Chromobacterium violaceum* CV026 and *P*. *aeruginosa* (Fu et al., 2013). Marine organisms such as macroalgae, sponges, corals, and invertebrates have constant microbial interactions, driving the evolution of chemical defences including QS-interfering compounds (Chen et al., 2019). Among these, macroalgae are particularly well studied. Halogenated furanones produced by *D. pulchra* inhibit virulence factor production in pathogenic bacteria (Manefield et al., 2001). This emphasizes the potential of marine-derived QSIs for use in aquaculture. Table 3 contains the details of QSIs from marine microorganisms and their applications.

**TABLE 3:** QSIs from marine microorganisms and their applications.

QSI/Derivative	Source	QS inhibitory activity	Application/Target	References
Kojic acid (γ-pyrone derivative)	Alternaria sp. (marine fungus)	Inhibited luminescence (E. coli pSB401)	LuxR interference	Li et al. (2003)
2,3-methyl-N-(2′-phenylethyl)-butyramide, N-(2′-phenylethyl)-isobutyramide	Halobacillus salinus C42 (sea grass)	Inhibition of QS-dependent violacein production in C. violaceum CV026 and green fluorescent protein (GFP) production in E. coli JB525	Antagonists competing with AHLs	Teasdale Margaret et al. (2009)
Meleagrin	Penicillium chrysogenium	Inhibited QS of C. violaceum CV017	FabI inhibitor + anti-QS	Dobretsov et al. (2011)
Diketopiperazines (cyclo (L-Pro-L-Phe), cyclo (L-Pro-L-Leu), etc.)	Marinobacter sp. SK-3	Inhibition of QS-dependent violacein production in C. violaceum CV017 and luminescence in reporter E. coli pSB401	QS interference in microbial communities	Abed et al. (2013)
Nocapyrones H, I, M (α-pyrones)	Nocardiopsis dassonvillei (Actinomycete)	Inhibited QS-controlled gene expression in C. violaceum, P. aeruginosa	Anti-QS metabolites	Fu et al. (2013)
Phenol, 2,4-bis(1,1-dimethylethyl)	V. alginolyticus G16 (seaweed)	Inhibited QS-mediated virulence factors and biofilm formation in S. marcescens	Anti-biofilm, antivirulence	Padmavathi et al. (2014)
MomL (AHL lactonase)	Muricauda olearia Th120	Degradation of short- and long-chain AHLs, Reduction of protease and pyocyanin production in P. aeruginosa	QQ enzyme, antivirulence	Tang et al. (2015)
Cyclo (Trp–Ser) (diketopiperazine)	Rheinheimera aquimaris QSI02	Reduction of QS-dependent violacein production in C. violaceum CV026. Reduction of QS-regulated pyocyanin production, elastase activity and biofilm formation in P. aeruginosa PA01	Biofilm and virulence suppression in P. aeruginosa	Sun et al. (2016)
Equisetin	Fusarium sp. Z10 (fungus)	Inhibited biofilm, swarming, elastase production, pyocyanin production in P. aeruginosa	Multi-system QS inhibitor (las, rhl, PQS)	Zhang et al. (2018)
Phenolic compounds (4-HBA, 4-HBAL)	Marine cyanobacteria (Leptolyngbya spp. MACC 32)	Reduced biofilm biomass, metabolic activity, motility and haemolytic activity. Decreased gelatinase production and EPS formationg LuxR/LuxP/LuxQ downregulation	QS inhibition via blocking AI-2 binding to LuxP. Reported to shrimp health	Saranya et al. (2025)

#### Plant-derived quorum-sensing inhibitors

Ethnobotanical studies of therapeutic herbs provide a foundation for identifying QSIs (Hsu et al., 2025). Higher plants, including vegetables, fruits, berries, cereals, and spices, are potential sources of QSIs, which may help reduce pathogen colonization and invasion. For instance, Cortex Moutan interferes with the QS system of *Pseudomonas fluorescens*. Bioactive compounds are typically extracted from dried plant material using solvents of varying polarity, such as water, ethanol, or ethyl acetate (Vattem et al., 2007). Preliminary screening for antibacterial and anti-QS activity is usually conducted using agar diffusion or micro-broth assays, enabling differentiation between direct antimicrobial effects and QQ activity. Some plant-derived QQ compounds and their anti-virulence effects against aquaculture pathogens are summarised in Table 4.

**TABLE 4:** Plant-derived QSIs and their applications.

Compound	Source	Target pathogen(s)	Mechanism	Observed effects	References
Cinnamaldehyde	Cinnamon (*Cinnamomum cassia*)	*Vibrio* species, including *V. parahaemolyticus*	Inhibition of AHL synthesis	Decreased virulence factor production; enhanced survival in shrimp models	Brackman et al. (2008)
Ajoene	Garlic (*Allium sativum*)	*P. aeruginosa*, *A. hydrophila*	Inhibition of LasR/LasI QS system	Reduced protease production, biofilm formation, and virulence	Jakobsen Tim et al. (2012)
Vanillin	Vanilla (*Vanilla planifolia*)	*A. hydrophila*	AHL degradation	Reduced biofilm formation and biofouling	Ponnusamy et al. (2013)
Quercetin	Various fruits and vegetables	*P. aeruginosa*	Inhibition of multiple QS targets	Broad-spectrum reduction in biofilm formation and virulence factor production	Ouyang et al. (2020)
Essential oil compounds	Various plants (e.g., thyme, clove, cinnamon, oregano)	*Vibrio* spp.	Antibacterial, anti-quorum sensing, anti-biofilm; often enhanced *via* nanoencapsulation	Reduced bacterial growth, QS-regulated virulence, and biofilm formation; reported to survival and health in aquatic animals; enhanced stability in aquaculture applications	Srinivasan et al. (2025)
Disulfide derivatives	Synthetic derivatives from natural products (allicin, ajoene, DADS, hordenine, cinnamic acid)	*P. aeruginosa*	Interaction with PqsR; disruption of QS system	Reduced virulence factor production, biofilm formation, motility; enhanced antibiotic efficacy; robust *in vivo* activity in *Galleria mellonella* model	Zhang et al. (2025b)

## Applications of quorum-quenching in aquaculture

### Quorum-quenching probiotics

Probiotics are microscopic but potent microbes that contribute to better health. The word itself is derived from the Greek words *bios* (life) and *pro* (for), which aptly describes their function: sustaining life. By restoring equilibrium to the gut microbiota, these beneficial microorganisms eventually enhance overall health. The use of probiotics with QQ capabilities is a promising alternative to antibiotics in aquaculture. By disrupting QS in pathogens, these quorum-quenching probiotics (QQPs) attenuate virulence and biofilm formation. Functional feeds containing QQPs, prebiotics, β-glucans, and bioactive compounds act through microbiome-derived metabolites, QS modulation, and omics-informed feed optimization. Currently, many QQPs have been studied. For example, *Bacillus velezensis* DH82, isolated from the deep-sea Yap Trench, demonstrated strong QQ activity against *Vibrio parahaemolyticus*, a major pathogen responsible for shrimp infections (Sun et al., 2022). Similarly, *B. velezensis* D-18 inhibited QS-regulated processes and biofilm formation in *V*. *anguillarum*, highlighting its role in fish health management (Monzón-Atienza et al., 2024). In another study, *Bacillus licheniformis* suppressed *S*. *Typhimurium* by inhibiting growth, motility, and biofilm development. At the MIC (0.5 mg/mL), it reduced immature (86.9%) and mature (66.9%) biofilms, downregulated the AI-2 system, and reduced EPS production (Peng et al., 2025). Rwezawula et al. (2025) isolated 24 indigenous bacteria from soilsamples. Among these isolates, 11 degraded N-hexanoyl homoserine lactone (HHL) and showed QQ activity. Among the QQ bacterial isolates, 8 were non-haemolytic. None of the isolates exhibited pathogenicity in *Artemia* nauplii, confirming their biosafety and probiotic potential (Rwezawula et al., 2025).

Fish gut-associated probiotics have also shown promising results. Two QQPs (*C. freundii* and *B. foraminis*) were isolated from the common carp gut. Dietary administration of these probiotics significantly reported to feed utilization, digestive enzyme activity, and fish gut microbiota (Abdulaziz et al., 2025). James et al. (2025) identified *B. cereus* and *B. velezensis* from mangrove sediments. These isolates exhibited antagonistic activity against *Aeromonas hydrophila*, *V*. *parahaemolyticus*, and *S. agalactiae*. Both *B. cereus* and *B. velezensis* exhibited enzymatic, antioxidant, and QQ properties, as well as the ability to degrade ammonia and nitrite (James et al., 2025). The impact of multi-strain QQPs on *Lates calcarifer,* fed QQPs containing *Bacillus thuringiensis* and *B. cereus,* resulted in enhanced growth, feed efficiency, antioxidant responses, immune gene expression, and survival against *Vibrio harveyi* (Mozanzadeh et al., 2025).

### Biofilm control through water treatment and biofilters

**FIGURE 5:**
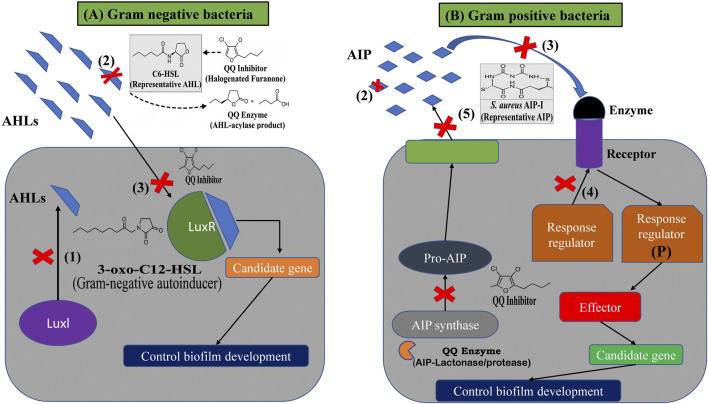
Mechanisms of QS inhibitors in biofilm control (concept adapted from Zhou et al., 2020): (1) Block AI synthesis, (2) Degrade/inactivate AIs, (3) Antagonize receptors, (4) Disrupt cascades, (5) Limit AI efflux (Prepared using Microsoft PowerPoint).

QQ bacteria can be utilized in biofilters to manage pathogenic biofilms in aquaculture systems. These bacteria degrade QS signals, preventing biofilm formation and reducing pathogen load in water systems (D’Aquila et al., 2024). In the last 2 decades, several QQ agents have been developed to suppress bacterial biofilm formation. These disrupt QS pathways by targeting (i) autoinducer (AI) signaling molecules, (ii) their receptors, or (iii) downstream signalling cascades (Zhou et al., 2020) (Figure 5). QQ enzymes such as AHL-lactonases and AHL-acylases degrade or neutralize AHLs in Gram-negative bacteria (Anandan and Vittal, 2019). Oxidoreductases such as BpiB09 and Hod modify AHLs or PQS, reducing their binding to receptors and limiting QS-regulated behaviours (Bijtenhoorn et al., 2011; Pustelny et al., 2009). Some QSIs act by binding to QS receptors, either competitively or non-competitively. Flavonoids such as naringenin and synthetic molecules like sitagliptin inhibit *Pseudomonas aeruginosa* LasR, suppressing pyocyanin and elastase production (Hernando-Amado et al., 2020; Abbas et al., 2020).

Biofiltration and recirculating aquaculture systems (RAS) have shown potential for sustainable water management, effectively removing pollutants, pathogens, and heavy metals (Das and Mishra, 2025). Substrate selection in RAS, such as PVC ecological nests, stabilizes water quality, enhances growth of *Babylonia areolata*, and limits antibiotic resistance gene accumulation (Zhang et al., 2025a). Macrophyte extracts, such as *Typha domingensis*, inhibited QS and biofouling by the invasive golden mussel (*Limnoperna fortunei*) (Morales et al., 2024). *Candida* sp. SW4-6, isolated from shrimp pond water, effectively reduced nitrite and ammonia concentrations during laboratory trials (Li and Liu, 2025). These studies collectively emphasise microbial, ecological, and substrate-based strategies for enhancing water quality and sustainability in aquaculture systems.

### Modulation of host immune response

The immunomodulatory and growth-promoting capabilities of probiotic bacteria are well established. Beyond direct effects on pathogens, QQPs can also influence host immune function through multiple mechanisms, potentially enhancing disease resistance and health status (Tyagi et al., 2024; Yousuf et al., 2023). Supplementation of feed with QQPs *B. thuringiensis* QQ1 and *B. cereus* QQ2 resulted in enhanced innate response in Asian seabass (*Lates calcarifer*). Even in the absence of pathogen challenge, bactericidal, antioxidant and respiratory burst activities in QQPs-fed fish groups were significantly (Ghanei-Motlagh et al., 2021a). Another study by the same research group also reported significantly higher growth and protection against *V. harveyi* challenge in *L. calcarifer* (Ghanei-Motlagh et al., 2021b). Dietary administration of QQP *B. velezensis* DH82 mitigated the harmful effects of *V. parahaemolyticus* pathogen on health status and immune response of *Litopenaeus vannamei*. Tissue imaging data also showed that *V. parahaemolyticus-*induced intestine and muscle damage in shrimp was ameliorated by DH82 (Sun et al., 2022). These studies show the positive impact of QQPs on the health status, growth and disease resistance of fish and shellfish.

### Potential synergies with conventional treatments

The integration of QQ approaches with conventional disease management strategies may result in significant synergistic effects. This synergy not only enhances treatment efficacy but also potentially reduces antimicrobial use (Sun et al., 2021). In bacterial biofilms, the extracellular polymeric substance (EPS) serves as a physical barrier, restricting the entry of antimicrobial drugs. Poor drug penetration into the innermost layers results in the development of drug resistance in microbes due to continuous sub-lethal dose exposure. The efficacy of antimicrobial compounds against pathogens could be significantly enhanced through QQ-mediated disruption of biofilms. Combined treatment with QQ quercetin and antibiotics at sub-MIC doses resulted in a synergistic effect, with ≥80% reduction in biofilm formation by an MDR clinical isolate of *P. aeruginosa* (Vipin et al., 2020). Combined application of the QQ enzyme Est816 and the antibiotic minocycline increased the sensitivity of *Aggregatibacter actinomycetemcomitans* biofilms to minocycline. This synergistic effect also increased the eradication rate of the bacterial pathogen (Wang et al., 2024). These studies highlight the synergy between QQ and conventional antimicrobials in pathogen control.

## Challenges and limitations

### Specificity of quorum-quenching molecules

The specificity of QQ molecules is a major challenge to their broad-spectrum applicability to control bacterial pathogenicity. In a complex microbial environment having multiple potential pathogens, QQ enzymes and molecules may not effectively inhibit all the QS signaling molecules. For example, AHL-acylases generally show higher activity against long-chain AHLs, and their activity is limited against short-chain AHLs. This signal-specific efficacy creates potential blind spots in pathogen control strategies (Utari et al., 2017; Kumar et al., 2022; Talagrand-Reboul et al., 2017). Implementing the QQ strategy is also complicated by the diversity and redundancy of QS signal molecules in pathogenic bacteria. Many bacteria have multiple parallel QS circuits that may operate independently or integrate with one another through complex regulatory networks. For example, *V*. *harveyi* employs three distinct QS systems (HAI-1, AI-2, and CAI-1) converging on a shared phosphorelay cascade (Waters and Bassler, 2006). Thus, a QQ molecule targeting a single signal type may not fully suppress virulence, as alternative pathways remain available.

### Development of resistance against QQ molecules

Due to their non-lethal mode of action, quorum-quenching techniques are frequently thought to be less likely to cause resistance; nevertheless, new research indicates that adaptive responses and resistance-like mechanisms may still occur. However, recent studies suggest that bacteria are emerging with resistance mechanisms to counter QQ interventions. Some of the common mechanisms are listed below (Krzyżek, 2019; Babaniyi et al., 2023; Maeda et al., 2012).

- To overwhelm the QQ molecule, overproduction of the QS signal molecule may occur in pathogenic bacteria
- The required threshold concentration of signal molecules to initiate the QS may be reduced
- QS signal molecules may undergo structural modifications, resulting in reduced susceptibility to degradation by QQ enzymes
- Mutations in QS receptors may allow these proteins to maintain functionality even at decreased signal molecule concentrations.
- Alternative signal pathways in pathogenic bacteria may evolve to bypass the targeted systems

### Environmental and biosafety issues

During the implementation of QQ strategies, environmental and biosafety issues must also be considered. Although the QQ approach is more eco-friendly than conventional antimicrobials, the potential impact of QQ compounds on non-target organisms is a primary concern. A broad-spectrum QQ approach may also affect the beneficial bacteria in the environment. Besides virulence factor production and pathogenicity, QS also plays important roles in symbiotic relationships, biochemical properties, and environmental adaptations in microbial communities (Rosier et al., 2021; Hartmann et al., 2024). Disruption of these important processes by QQ may cause unintended disturbance in the aquatic ecosystem.

### Production scalability and commercial viability

The transition of the QQ strategy from laboratory experiments to commercial field applications is a major challenge. Cost is the major limiting factor for the direct use of QQ enzymes in aquaculture. Though the enzyme yields can be reported to using the recombinant systems, cost-efficient production, purification, and stabilization at a large scale is challenging due to narrow profit margins in the aquaculture sector (Rudge and Ladisch, 2020). For QQ compounds of plant or marine origin, supply chain limitations, such as inconsistent availability and quality standardization, are the major issues. Synthetic analogs of natural QQ compounds are a potential option, but they may face higher regulatory hurdles and development costs.

## Future prospects and research needs

Identification and characterization of novel QQ compounds from diverse sources is crucial to improve fish health in sustainable aquaculture. Aquatic environments, especially marine ones, are rich reservoirs of QQPs, enzymes, and other molecules that are active against QS signals produced by pathogenic bacteria. Advancements in synthetic biology and protein engineering could be utilized to develop QQ enzymes/compounds having higher activity and stability. Structural analogues of natural QQ compounds could also be developed to inhibit pathogen communication systems. QQ compounds/enzymes from microbes of extreme environments, such as thermophiles, halophiles, and deep-sea bacteria, may be better adapted to challenging aquaculture conditions. Advanced delivery systems, including biodegradable polymers, hydrogels, and microencapsulation, can protect QQ compounds from degradation and prolong their activity. Besides, pH- or enzyme-triggered or nanoparticle carrier systems can release the QQ compounds at the precise sites of pathogen colonization.

The paradigm change from reductionist approaches to holistic systems biology frameworks driven by multi-omics integration is crucial for the future of biological and biomedical research (Saleem et al., 2026). The dynamic and interconnected biochemical processes that underlie complex illnesses and broader biological features can be captured by researchers through the simultaneous analysis of genomics, transcriptomics, proteomics, metabolomics, and epigenomics. By analyzing the interactions between metabolic pathways, protein networks, and epigenetic landscapes, this comprehensive molecular image enables precision health treatments and the identification of hitherto hidden therapeutic vulnerabilities. Additionally, a high-resolution understanding of how deep cellular regulation affects coordinated neuroimmune and metabolic systems is made possible by advanced multi-omics integration, such as integrating expression quantitative trait loci (eQTL) with genome-wide association studies (GWAS) and single-cell RNA sequencing (Shan et al., 2026). These integrated frameworks have the potential to transform biological prediction models, find new biomarkers, and accelerate the conversion of systems-level knowledge into practical, real-world applications as computational modeling and artificial intelligence continue to address persistent issues with data heterogeneity and standardization. The technologies enable a deeper understanding of virulence regulation, metabolic adaptations, and resistance mechanisms. Pathogen-specific and community-level effects of QQ strategies can be inferred from metagenomic and meta-transcriptomic analyses. These studies ensure that beneficial microbes are preserved and pathogens are suppressed during QQ application. In advanced farming systems such as RAS, Biofloc, and aquaponics, the use of chemical antimicrobials is detrimental to the overall ecosystem. Thus, integrating QQ with these farming practices ensures disease control while maintaining ecological balance. Artificial intelligence (AI)-based real-time monitoring and smart delivery systems could enable adaptive dosing according to pathogen load and environmental conditions. Finally, standardized protocols for QQ assessment, application, and monitoring are essential for reproducibility, field evaluation, and regulatory compliance in sustainable aquaculture.

## Conclusion

QQ is an innovative emerging health management approach that targets bacterial communication systems. QQ attenuates bacterial pathogenicity without significantly affecting their community composition. Enzymatic and non-enzymatic QQ compounds from microbial, plant, and marine sources offer multiple intervention points for QQ in aquaculture production systems. The use of QQ through QQPs, feed additives, and water treatment has led to reduced disease outbreaks, reported to growth performance, and reduced reliance on conventional antimicrobials. Although challenges related to specificity, potential resistance, environmental impacts, and scalability remain, integrating QQ with sustainable aquaculture practices can enhance productivity and reduce environmental degradation.
